# Literature Review: Theory and Application of In-Line Inspection Technologies for Oil and Gas Pipeline Girth Weld Defection

**DOI:** 10.3390/s17010050

**Published:** 2016-12-28

**Authors:** Qingshan Feng, Rui Li, Baohua Nie, Shucong Liu, Lianyu Zhao, Hong Zhang

**Affiliations:** 1 School of Mechanical and Transportation Engineering, China University of Petroleum, Beijing 102249, China; hzhang@cup.edu.cn; 2 PetroChina Pipeline Company, Langfang 065000, China; lyzhao@alum.imr.ac.cn; 3 School of Automation Science and Electrical Engineering, Beihang University, Beijing 100191, China; 4 School of Materials Science and Energy Engineering, FoShan University, Foshan 528000, China; niebh@petrochina.com.cn; 5 Institute of Disaster Prevention, Sanhe 065201, China; liushucong@cidp.edu.cn

**Keywords:** pipeline girth weld, defect, in-line inspection, magnetic flux leakage inspection, ultrasonic inspection, EMAT

## Abstract

Girth weld cracking is one of the main failure modes in oil and gas pipelines; girth weld cracking inspection has great economic and social significance for the intrinsic safety of pipelines. This paper introduces the typical girth weld defects of oil and gas pipelines and the common nondestructive testing methods, and systematically generalizes the progress in the studies on technical principles, signal analysis, defect sizing method and inspection reliability, etc., of magnetic flux leakage (MFL) inspection, liquid ultrasonic inspection, electromagnetic acoustic transducer (EMAT) inspection and remote field eddy current (RFDC) inspection for oil and gas pipeline girth weld defects. Additionally, it introduces the new technologies for composite ultrasonic, laser ultrasonic, and magnetostriction inspection, and provides reference for development and application of oil and gas pipeline girth weld defect in-line inspection technology.

## 1. Introduction

Oil and gas pipelines are the main arteries of the national economy; pipeline safety is closely related to people’s lives, social and economic development. Although pipeline transport is the safest and most reliable means of transporting for flowing mediums, leakage occurs every year due to welding defects, corrosion and local damage, resulting in severe losses or personal injury and even death. Among them, due to the limited technical level of the construction period, on-site welding construction, operating environment and other factors, girth weld has always been a weak part of the pipeline, which made girth weld cracking one of the main failure modes of oil and gas pipelines. Among the failure accidents in 2010–2012, as released by the US Department of Transportation Pipeline and Hazardous Materials Safety Administration, up to eight accidentswere caused by the girth weld cracking of gas transport pipelines (regardless of perforation caused by girth weld corrosion), three of the accidents occurred in the new commissioning pipeline [1]. As revealed in the survey on the causes of eight recent girth weld cracking accidents of new pipelines in China, obvious welding defect was found in six accidents (the other two accidents resulted from too high external load but also involved girth weld defects), and girth weld cracking in seven accidents started from the internal wall of pipe [2]. The girth weld cracking in oil and gas pipelines has the characteristics of long crack length and large leakage, which poses a serious threat to the safety of surrounding personnel, environment and pipeline transportation. Considering the risk of girth weld cracking of oil and gas pipelines, there are strict technical and managerial requirements for field welding during pipeline construction, also the visual, X-ray and ultrasonic inspections must be conducted. However, still there are lots of defective girth welds. For this reason, pipeline operation companies, both domestic and overseas, developed the pipeline integrity management concepts and system. Inspection companies use all kinds of advanced inspection technologies to inspect and identify pipeline defects, to accurately determine the type of defects and quantify the size [3], in order to conduct the safety evaluation, repair or maintain defective pipelines and guarantee the intrinsic safety of oil and gas pipelines. Those methods include three-axis high-resolution magnetic flux leakage (MFL) inspection, liquid ultrasonic crack inspection, electromagnetic acoustic transducer (EMAT) inspection, and remote field eddy current (RFEC) inspection technologies, in a bid to realize high detection accuracy of girth weld defects and repair the defects in advance. This paper analyzes the characteristics of long-distance transport pipeline girth weld defects, and summarizes the inspection principle, defect signal identification, defect sizing method and inspection reliability of existing girth weld defect in-line inspection technologies systematically, which include MFL inspection, liquid ultrasonic inspection, EMAT inspection and RFDC inspection for oil and gas pipeline girth weld defects. Moreover, it introduces the new technologies for composited ultrasonic, laser ultrasonic, and magnetostriction inspection, and provides reference for the development and application of oil and gas pipeline girth weld defect in-line inspection technology.

## 2. Oil and Gas Pipeline Girth Weld On-Site Inspection

**Figure 1:**
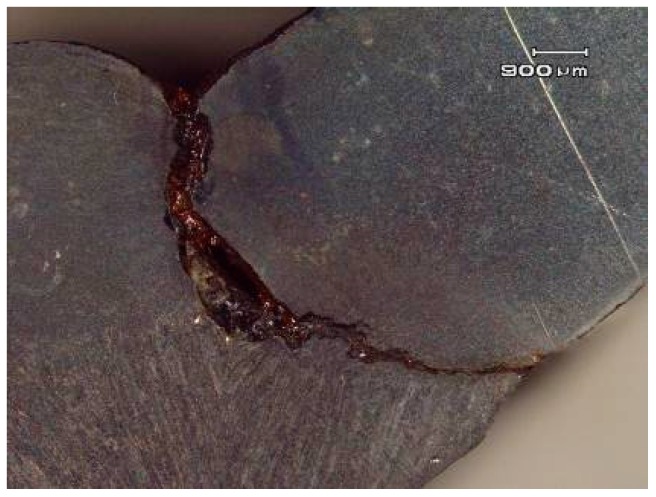
Lack of fusion and crack defects of welding.

**Figure 2:**
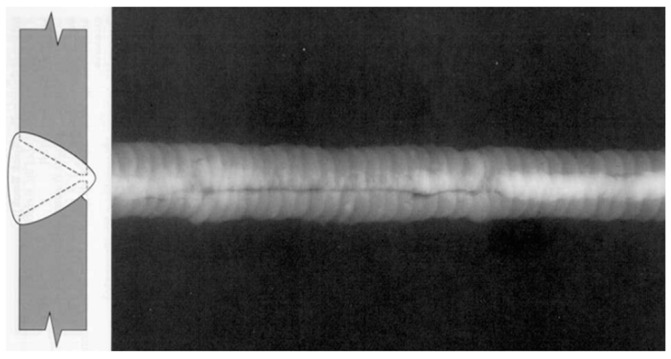
X-ray inspection of an undercut defect inside the weld.

The girth weld defects of oil and gas pipelines can be roughly classified into planar defects, volumetric defects and irregular shape defects. Planar defects include incomplete penetration, lack of fusion, crack and undercut, etc. Incomplete penetration refers to the gap between base materials caused by insufficient arc welding; lack of fusion results from the insufficient fusion and bonding between weld metal and base material or between weld metals under several passes. The former is also known as lack of side fusion, while the latter is called lack of inter-run fusion. A crack appears in the weld during or after cooling under the welding stress or the residual stress of formation. The lack of fusion and crack defects of pipeline weld are shown in Figure 1. An undercut is a notch left when arc seam edges are not supplemented by filler metal after they are molten. Stress concentration may easily happen at an undercut, and a crack may occur at undercut after a load is applied. Figure 2 presents the profile of an internal undercut during X-ray inspection.

**Figure 3:**
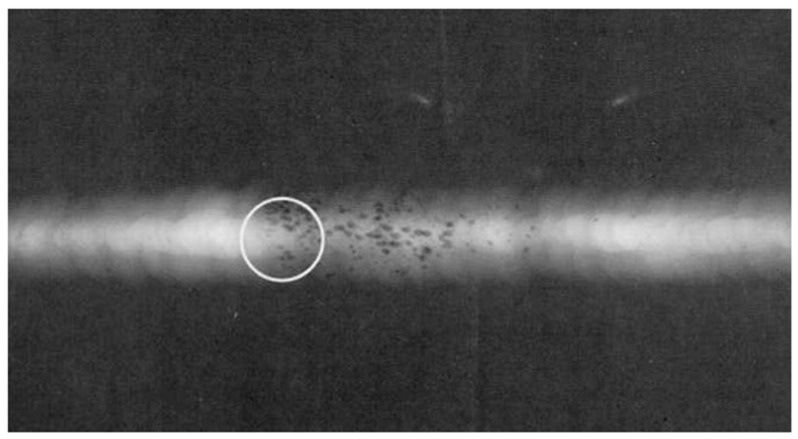
X-ray inspection of weld porosity.

**Figure 4:**
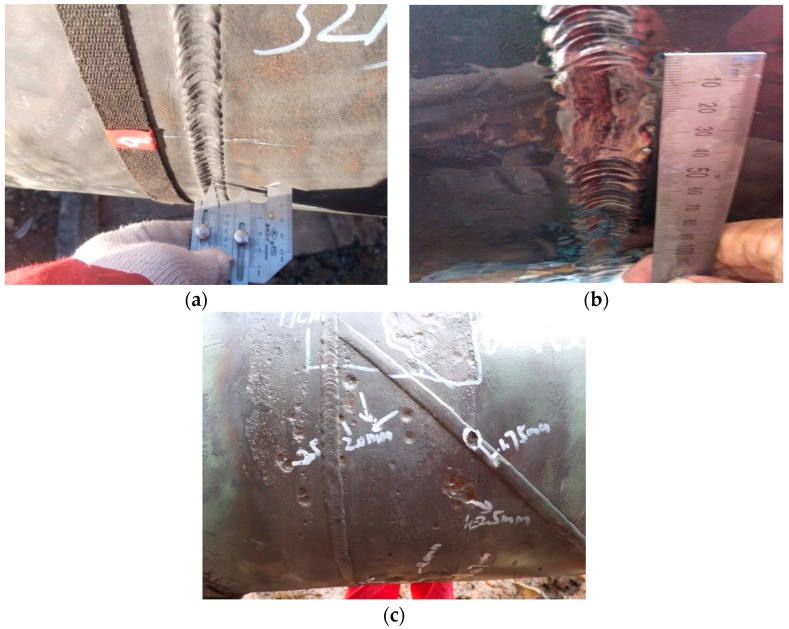
Lack of girth weld metal: (**a**) underfill; (**b**) bevel grinding; (**c**) weld corrosion.

Volumetric defects include porosity, slag inclusion, underfill, bevel grinding and weld corrosion, etc. Porosity is air pores formed when the air generated in the process of welding fails to escape but remains inside weld metal. Slag inclusion occurs when the slag of welding flux fails to float out of the weld pool and is trapped in the weld pool during the melting–cooling process. Porosity and slag inclusion often occur inside weld metal. Figure 3 presents the profile of weld porosity during X-ray inspection. Underfill, bevel grinding and weld corrosion is caused by the lack of girth weld metal, and their typical profiles are shown in Figure 4.

**Figure 5:**
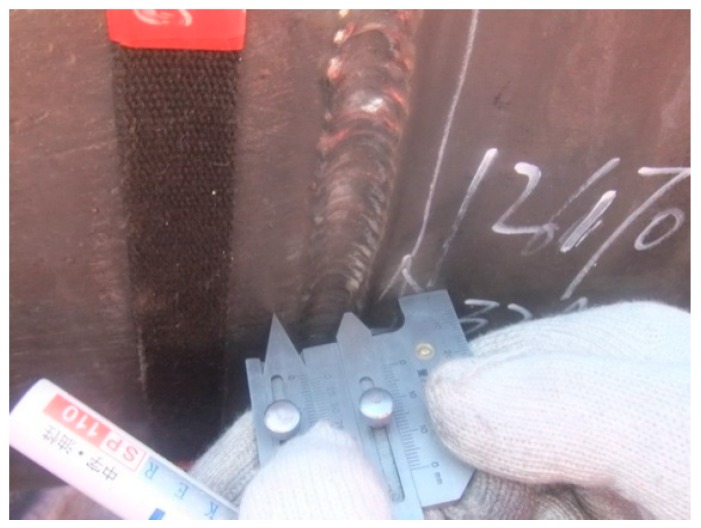
Profile of a mismatched weld joint.

Irregular shape defects contain an irregular weld outline, and mismatched weld joint, etc. A mismatched weld joint means that the two edges of the pipeline are mismatched during welding (as shown in Figure 5); this could reduce effective wall thickness, which may cause extra bending stress. Irregular weld outline is easily caused by construction and other factors, e.g., difficult welding for girth weld at the bottom of the pipeline.

At present, domestic and overseas accidents of oil and gas pipeline girth weld cracking are mostly caused by a crack, incomplete penetration, lack of fusion, and a sharp undercut. These planar defects have a high degree of stress concentration, which significantly lowers the bearing capacity of pipelines, so nondestructive testing of oil and gas pipelines should focus on these kinds of girth weld defects.

**Figure 6:**
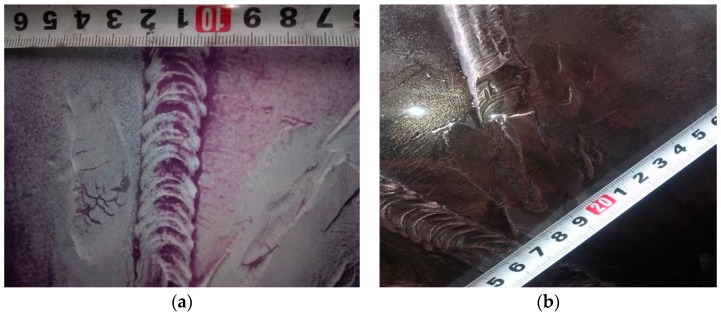
Inspections for external defects of pipeline girth weld: (**a**) dye penetrant; (**b**) magnetic particle.

For pipeline girth weld, external defects can be inspected by employing magnetic particle or dye penetrant; internal defects can be mainly revealed through X-ray or ultrasonic inspection [4]. Magnetic particle inspection utilizes the irregular magnetic lines on the defect position of the magnetized weldment to locate the defect. It is applicable to magnetic metal weldment of varying thickness, which must have a smooth surface, and it is able to discover the 1~2 mm cracks on and under the surface. Dye penetrant inspection could provide a color image of the defect shape. A highly penetrating color liquid could enter the surface defect of a work piece, removing the excess liquid on the surface, and then applying the developer that absorbs the liquid. This method can be used to inspect the weldment of any material and thickness, but it requires the surface roughness Ra = 3.2 μm. It is able to detect any surface defect with the width no less than 0.01 mm and the minimum depth 0.03~0.04 mm. The magnetic particle and dye penetrant inspections for external defects of pipeline girth weld are presented in Figure 6.

Before ultrasonic inspection appeared, X-ray radiography was the main way to reveal the defects hidden in weld. It detects a defect based on the variation of X-ray beam passing through due to different absorptions of the defect and its surrounding metal. X-ray absorption depends mainly on the density of the material, so X-ray radiography is a very effective way to detect the volumetric defects (e.g., slag inclusion or porosity) (Figure 3), but it is not always effective to deal with the planar defects (e.g., dense cracks or lack of fusion) at any direction inside a thick metal piece. X-ray radiography is less reliable than automatic ultrasonic technology, and unable to accurately measure the size of a crack penetrating the wall thickness (i.e., crack depth). Additionally, the regular inspection with X-ray radiography faces such problems as obscure film, low contrast and poor sensitivity, which affect the qualitative and quantitative accuracy of defects, since the oil or medium inside the pipeline causes the absorption and scattering of the X-ray. Therefore, it is necessary to drain and replace the medium inside the pipeline or make corresponding improvements to the X-ray inspection technology [5].

**Figure 7:**
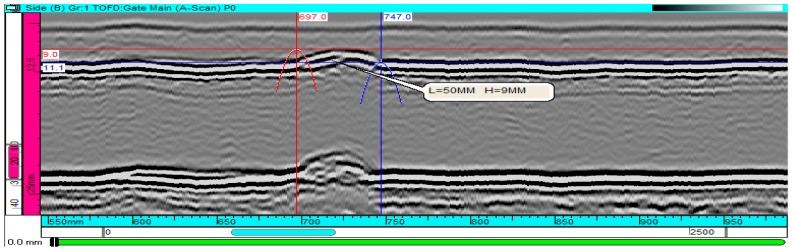
Time of flight diffraction technique (TOFD) inspection of the linear defect at the root of the weld.

Ultrasonic inspection is presently an important nondestructive testing technology for pipeline girth weld defect inspection. Ultrasonic waves could be scattered by planar and volumetric defects, and the echo signals from defects can be utilized to identify two such types of defects, measure the dimension and depth of defects and judge the nature of defects. Regular ultrasonic manual or fully-auto inspection has been widely applied in the detection of pipeline weld imperfections. Along with the increasing requirements for the ability to inspect the small weld defects, some advanced ultrasonic inspection technologies have been developed, such as phased array ultrasonic inspection [6] and time of flight diffraction technique (TOFD) [7]. Among them, TOFD utilizes a pair of transmitter and receiver probes arranged at both sides of weld, and employs the tilted wedge for the generation of a longitudinal wave. Following the principle of wave diffraction, it can ideally realize the detection and sizing of weld defects without being affected by factors such as sound beam angle, detection direction, and surface roughness of the defect [7]. TOFD does not rely on the reflective energy of the defect to identify it, which is an innate weakness of regular ultrasonic inspection, so it has a wide application prospect in pipeline girth weld defect inspection. Figure 7 presents the inspection of girth weld defects through TOFD after excavation of the pipeline, which reveals the linear defect at the root of the weld.

Common on-site weld defect inspection technologies are applied in the girth weld defect inspection during pipeline construction or after pipeline excavation. However, advanced oil and gas pipeline in-line inspection technology is still needed to inspect the defects of long-distance transport pipelines in operation, which have multiple girth welds, so as to realize the overall girth weld defect inspection of long-distance transport pipeline. For nearly three decades, multiple technologies have been developed and applied in the inspection of oil and gas pipeline defects, including corrosion, stress corrosion and crack, such as, high-resolution MFL inspection [8], liquid ultrasonic inspection [9] and natural gas pipeline electromagnetic acoustic transducer (EMAT) crack inspection [10], but as for the girth weld defect inspection, it is still in the exploration and development stage. Due to the irregular shape and profile of girth weld, the accuracy of inspection equipment is affected significantly. Also, long-distance oil and gas pipelines have narrow girth welds (normally 10~20 mm), which allow a very short time window for the action of inspection equipment, and make it very difficult to inspect defects. Girth weld defect inspection has always been a difficult problem around the world. Along with the continuous development of in-line inspection technology, the research and development of girth weld defect inspection technology has started. Domestic and overseas pipeline inspection companies as well as pipeline operators have been working closely to improve the existing oil and gas pipeline in-line inspection technologies and equipment and engage in the research and application of pipeline girth weld defect inspection technologies.

## 3. Oil and Gas Pipeline Girth Weld Defect In-Line Inspection Technology

### 3.1. Three-Axis High-Resolution Magnetic Flux Leakage Inspection

#### 3.1.1. Inspection Principle and Signal Analysis

**Figure 8:**
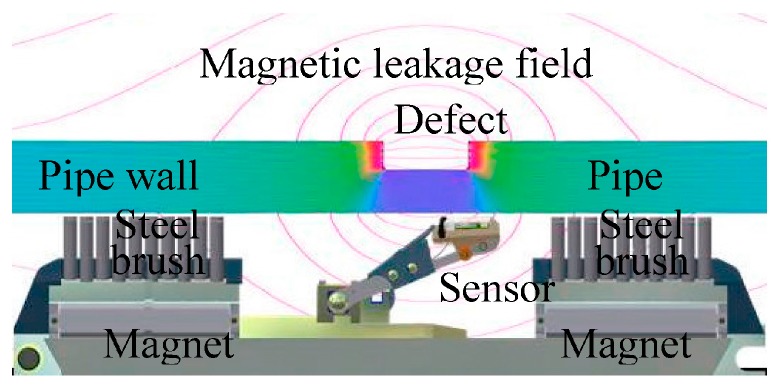
Schematic diagram of the magnetic flux leakage in-line inspection principle.

The principle of defects detecting and sizing of MFL inspection equipment is based on the varying magnetic lines of defects on the pipeline (Figure 8); it could obtain the location, type, shape, dimension and other information of defects through the identification and judgment of MFL data. Three-axis high-resolution MFL in-line inspection utilizes the three-direction sensors inside the existing sensor to detect the size of the magnetic field in three directions. Therefore, it could measure the axial, circumferential and radial MFL data to determine the three-dimensional magnetic leakage field vector, in order to improve the inspection accuracy of defects [11].

**Figure 9:**
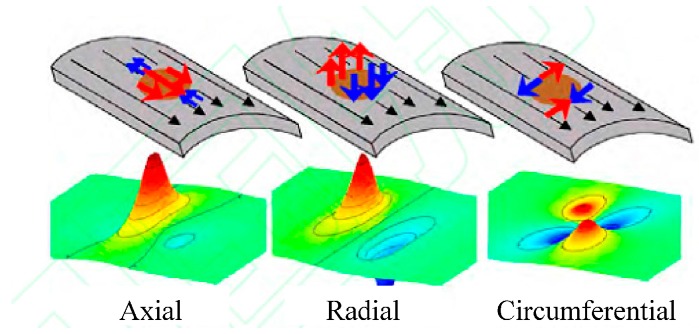
Three-axis signals of typical metal loss defect.

The axial magnetic leakage field component of a metal loss defect is a typical uni-polar waveform, and its amplitude is highly related to defect depth, but the axial signal has nonlinear change in the directions of width and length, so the length and width of defects can only be estimated based on the range of signal deformation, causing very low accuracy and reliability. The radial signal component shows bipolar sine waveform with equal amplitude of peak and trough, which is highly correlated with defect depth. The gap between peak and trough can greatly reflect the length of the defect. The circumferential signal shows the positive and negative polarities in both radial and circumferential directions, and visually reflects the width and length of the defect (Figure 9). This technology can effectively detect and size the defects of long-distance transport pipelines, such as metal loss (corrosion and mechanical scratch) [12,13,14,15,16]. Additionally, PetroChina Pipeline Company (Langfang, China) has, through cooperation with GE Oil & Gas Pipeline-inline-inspection Solutions (GE PII, Newcastle, UK), applied the three-axis high-resolution MFL technology in the inspection and sizing of volumetric defects in the spiral weld of long-distance transport pipelines, so as to successfully eliminate the problem of spiral weld cracking of the aged pipes in Northeast China [17,18,19,20]. It is important to note that inspection is very difficult due to the dramatic decrease of magnetic flux leakage outside the pipe wall, along with the increase of wall thickness and inspection speed [21].

**Figure 10:**
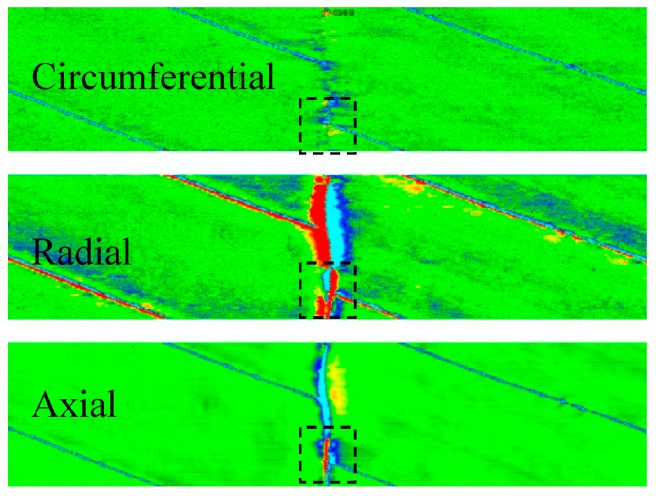
Three-axis magnetic flux leakage (MFL) signals of a girth weld defect.

In recent years, Kinder Morgan (Houston, TX, USA), TransCanada (Calgary, AB, Canada) and PetroChina Pipeline Company (Langfang, China) are engaging in the application of MFL in-line inspection technology in the inspection and research of girth weld defects [2]. As revealed in the research conducted by Wang Fuxiang et al. [22], a girth weld defect with certain metal loss shows signal features similar to those of metal losses including corrosion. For volumetric girth weld defects, including lack of fusion, incomplete penetration and underfill, the axial signal has a positive peak (red for positive and blue for negative) with two small negative peaks; radial signal has a negative peak and a positive peak; and circumferential signal has two positive peaks and two negative peaks within a nearly rectangular scope (Figure 10). On this basis, the researchers of PetroChina Pipeline Company (Langfang, China) have carried out the systematic comparison and classification of signals for girth weld defects, including lack of fusion, incomplete penetration, missing cap, mismatched weld joint and grinding, so as to provide the guidance for accurate identification of girth weld defects.

Efforts are still being made to study and explore the application of MFL in-line inspection technology in the inspection and sizing of girth weld crack defects. Liu et al. [23] built a three-dimensional model of cracks to explore the influence of crack parameters on MFL signals, including depth, width, inclination angle and crack gap, etc. As revealed in the research results, the inclination angle of the crack has an obvious impact on the amplitude of MFL. In the practical inspection, one-way inspection may result in missing a spot, and magnetic leakage fields may overlap each other when the gap between two cracks is less than 5 mm. In the studies on finite element simulation and traction test by Wang Fuxiang et al. [22] and Wang Ting et al. [24], three-axis high-resolution MFL inspection can only detect the defects with a large opening (>0.5 mm). Natalia et al. [25] proposed a new MFL tool designed to generate a field of excitation in the circumferential or transverse direction, and detect the axial cracks. Using this method, 19 out of 22 features, such as a crack in the electric resistance welding (ERW) were verified to be linear long-seam defects, which were subsequently repaired in a permanent manner.

#### 3.1.2. Defect Sizing Methods

At present, a lot of research work has been done in the field of signal analysis and quantitative analysis of three-axis high-resolution MFL. The main analysis methods include finite element analysis, neural network and so on.

(1) Finite Element Analysis

In the defect sizing of MFL inspection, the ANSYS modeling of finite element analysis is employed to study the relationship between geometric parameters and MFL signals for pipeline defects. Ding Zhanwu et al. [26] pointed out that for the defects of the same length, the depth of defect and the amplitude of the MFL signal are approximately linear. For the defects of the same depth, there is basically a linear relationship between the defect length and the width of the MFL signal. Through the combination of a finite element simulation model with a genetic algorithm, K.C. Hari et al. restructured the shape of a pipe defect [27]. Reza Khalaj Amineh et al. introduced the tangent component of a MFL signal to describe the direction, length and depth of surface crack defects [28]. Yang Lijian et al. built a MFL three-axis in-line inspection simulation model for pipeline, established the relationships of axial, radial and circumferential components of the magnetic leakage field with the defect size of the pipeline, and eventually developed the pipeline MFL in-line inspection equipment successfully through continuous efforts [29,30,31]. Additionally, domestic and overseas scholars studied the speed effect in the MFL inspection process through finite element simulation, and put forward the speed effect compensation method [32,33,34].

(2) Neural Network

The application of a neural network in approaching the functional relationship between geometrical parameters and MFL signals of a defect has become a hot topic of research, and has made great progress. Shen-Tun Li put forward the defect pattern recognition method for nondestructive testing based on fuzzy subset theory, and applied the radial basis function neural network in the interpolation calculation and non-linear approximation of a magnetic leakage field. Also, he created a radial basis function (RBF) network model and proposed the learning algorithm, so as to provide a feasible method for pipeline defect sizing [35]. R. Christen et al. proposed the practical algorithm based on model feature extraction and neural network for automatic defect detection in the MFL inspection [36]. Hwang Kyungtae [37], Ameet Joshi et al. [38], and Pradeep Ramuhalli et al. [39] recommended applying the wavelet neural network to simulate the relationships between geometric parameters and MFL signals of a defect, and put forward the inversion algorithm based on the neural network of the adaptive wavelet basis function, which could greatly restructure the defect outline when there is any noise.

With regard to the domestic studies on the neural network of a pipeline defect, Wang Taiyong et al. [40,41] employed the entropy spectral analysis method to characterize MFL signals, put forward the pipe defect sizing and identification technology based on the neural network of radial basis function, and also studied the sizing technique for the MFL inspection of oil and gas transport pipeline defects. Jin Shijiu et al. [42,43] applied the radial basis neural network and wavelet neural network to predict the nonlinear relationships between MFL signals and geometrical parameters of a defect. Yang Lijian et al. [44,45] utilized the finite element analysis method to study the relationships between MFL signals and defect parameters, and introduced the pipeline defect sizing and identification method based on the neural network and data fusion.

#### 3.1.3. Inspection Reliability

The three-axis high-resolution MFL inspection for the volumetric defects (corrosion and scratch) of long-distance transport pipelines has the dimensional accuracy of ±10%, and its confidence level exceeds 90%, so it can satisfy the regular needs of the inspection of volumetric defects in oil and gas pipelines [8,46]. Nevertheless, three-axis high-resolution MFL inspection is not a mature technology for the inspection and sizing of volumetric defects and crack defects in the pipeline girth weld. Through a traction test and field test, PetroChina Pipeline Company (Langfang, China) carried out the systematic assessment of three-axis high-resolution MFL inspection reliability [22,24]. The probability of volumetric defect inspection for long-distance transport pipeline girth weld was higher than 93%, while the defect depth was more than 98% within ±25% wall thickness (wt). The sizing of the depth for girth weld defects with a 3 mm and 5 mm opening was the most accurate. The sizing of circumferential length of defects was accurate and acceptable, but the sized length of the opening was much larger than the actual length. Nevertheless, it failed to detect five girth weld defects at the 5° skewed connection, and the depth of one girth weld defect with variable wall thickness was beyond the deviation ±20%. Furthermore, it was impossible to separate internal and external defects of a pipeline under the effect of weld.

In the reliability evaluation of simulated girth weld defect inspection for cracks, there were 102 defects, including seven defects to the report threshold of MFL in-line inspection and 39 defects (accounting for 38.2%) indicated in the MFL signal. The accuracy of all defect depths was within 25% wt, but it required the defect width at least 0.25 mm. Three-axis high-resolution MFL inspection technology could poorly detect girth weld crack defects and had poor accuracy of defect sizing, so it had some limitations. It should be noted that varying wall thickness, skewed weld and mismatched weld joint affect the signal identification of the girth weld defect significantly, and it is now very difficult to differentiate the signals of girth weld defects and mismatched weld joints.

Based on the girth weld defect signal of three-axis high-resolution MFL inspection for a crude oil pipeline (X65), the excavation examination was conducted at those 32 defects in a focused manner, including lack of fusion/incomplete penetration at eight positions, missing cap at 10 positions and other defects at 14 positions. The defect depth deviation was ±6%, and the confidence level was 90%. A gas pipeline was cut for girth weld defects at 13 positions, including lack of fusion at 11 positions, which were all detected through MFL4, but crack defects at two positions (with an opening of 0.3 mm) were not detected. The deviation of the defect depth for lack of fusion in girth weld during MFL4 in-line inspection was ±15%. The defects with the opening width of 0.3 mm could not be detected in MFL4.

MFL inspection has some limitations for the crack defects of girth weld, but it does not need a coupling agent, and is able to detect the girth weld defects of gas pipelines as well as many types of defects. Hence, it is comprehensively concluded that this technology is the first choice for baseline screening of girth weld defects.

### 3.2. Ultrasonic Inspection for Oil Transport Pipeline Girth Weld Cracks

Ultrasonic crack inspection equipment can identify tiny cracks [47,48], stress corrosion cracks [49] and fatigue cracks [50], etc. This technology has very high accuracy of inspection with regard to crack defects, but it must rely on the coupling between liquid medium and pipe wall, so it is difficult to apply it in the gas pipeline.

#### 3.2.1. Inspection Principle and Signal Analysis

**Figure 11:**
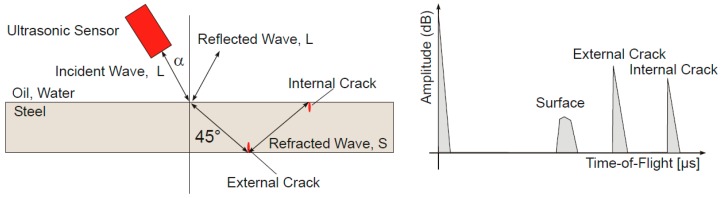
Principle of ultrasonic measurement.

Crack ultrasonic inspection equipment follows the principle of ultrasonic measurement based on pulse echo time technology. In the common ultrasonic nondestructive testing technology, 45° wave technique is successfully utilized to achieve the axial crack ultrasonic inspection of pipelines. This technology employs ultrasonic sensors to transmit the ultrasonic waves into pipe wall at a specified incident angle. When ultrasonic waves encounter any reflective characteristics (e.g., edge, crack), some ultrasonic waves are reflected back to the original position (Figure 11). By assessing the time of flight of the echo signal and all kinds of signal characteristics, it can predict the positions and sizes of these characteristics. For a circumferential defect, its length can be determined based on the number of sensors receiving the echo signal.

**Figure 12:**
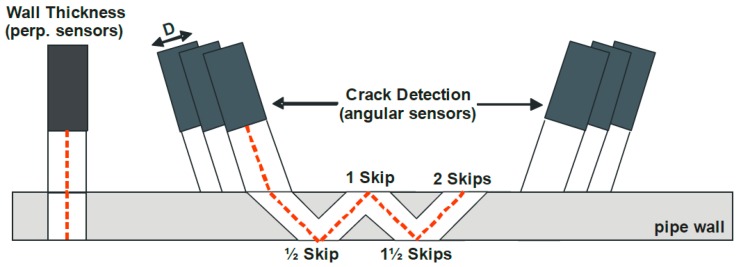
Principle of sensors in ultrasonic inspection equipment.

At present, this technology has not been applied in the practical girth weld defect inspection of pipelines in other countries. Through cooperation with GE PII, PetroChina Pipeline Company (Langfang, China) has carried out a series of studies [51] and improved the applicability of equipment to circumferential defects by resetting the direction of sensors. The inspection for circumferential crack defects of girth weld has a different configuration of sensor carrier and data recognition from traditional axial crack inspection. Sensors can be divided into upstream and downstream directions. In addition, each track is equipped with two perpendicular ultrasonic sensors to measure the wall thickness and identify characteristic location. External cracks can be identified by half or one-and-a-half skip reflection, while internal cracks depend on one or two skips for identification, as shown in Figure 12.

**Figure 13:**
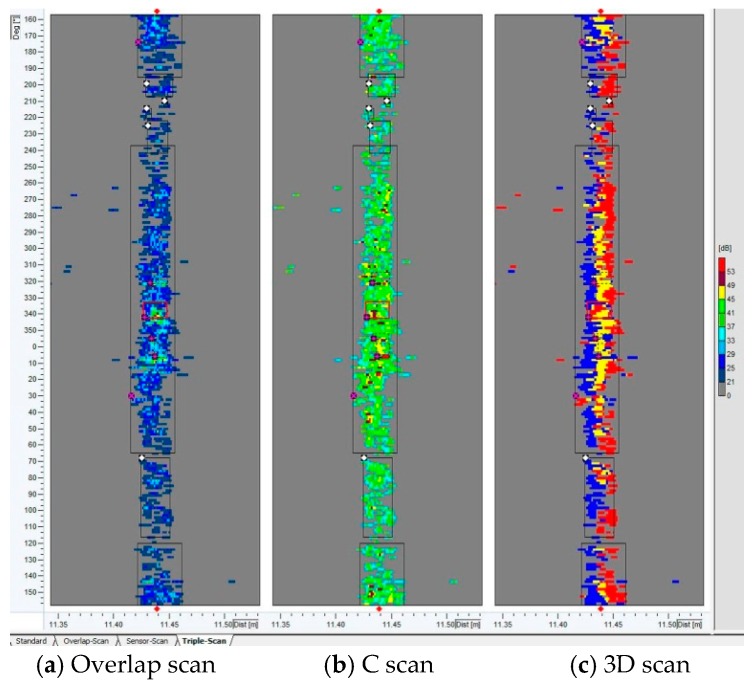
Signal characteristic of a premade girth weld defect during ultrasonic in-line inspection. (**a**) Overlap scan; (**b**) C scan; (**c**) 3D scan.

During identification, the girth weld defect is comprehensively analyzed based on the signals of C-scan view, overlap view, and 3D view (Figure 13). Figure 13a is an overlap view, which represents all recorded readings at the same position and stands for the number of sensors recognizing the characteristic. Figure 13b is a C scan view, representing the strength of the reflected signal. The higher the strength of the reflected signal, the darker the color is. Figure 13c is a 3D view, in which the blue area stands for the upstream edge of the weld detected by the upstream sensor (blue), and the red area is the downstream edge of the weld detected by the downstream sensor (red). The yellow area is the center of the weld, where upstream and downstream sensors coincide. If the position of color (red or blue) in the signal is different from the normal weld characteristic, there may be some abnormalities. Generally, if there is any abnormality at the same position in the C scan view, overlap view and 3D view, the position is recognized as the linear defect characteristic.

#### 3.2.2. Defect Sizing Method

Theoretically, the physical measurement of defect depth is conducted based on the number of sampling points. In the experiment process, the zero position of the sensor and the difference between the acoustic velocities of ultrasonic waves in the base metal and weld have a significant influence on the accuracy of the experiment. Hence, it is necessary to calibrate the acoustic velocity during the experiment, and determine the zero position of the sensor, which is a very complicated process. The neural network intelligent method can simplify the experiment process, and be applied in the defect sizing of long-distance transport pipelines. Masnata and co-workers [52] made 135 artificial defects including crack and slag inclusion, and utilized the Fisher method to process the parameters obtained through ultrasonic inspection. They selected 16 values of waveform, including rise time, fall time, and continuous response as the input of the neural network, and accurately realized the classification and identification of three types of defects. Reference [53] analyzed the frequency domain of echo signal, and characterized copper and aluminum to identify and study the category of these materials by means of a feed-forward artificial neural network. Zhao Jing et al. [54,55] characterized the echo signal based on different energy values when the crack signal is contained in the ultrasonic echo signal. By characterizing the entropy after wavelet packet analysis, the support vector machine is utilized to select the suitable kernel function for automatic identification and classification of corrosion cracks. Yang Zhuoran [56] particularly performed the analysis and modeling of crack echo signals obtained; employed the wavelet transformation for delicate processing; and created the in-line inspection position model for cracks. Chen Guohua et al. [57] further explored the method and feasibility of intelligent recognition for crack defects based on the wavelet extraction of ultrasonic echo signal defect characteristics, the structural parameters of the neural network and the training and testing network, etc.

#### 3.2.3. Inspection Reliability

Liquid ultrasonic inspection technology achieves satisfying results in the inspection and defect sizing of axial cracks (stress corrosion crack and fatigue crack) in long-distance transport pipeline, so as to effectively lower the risk of an axial crack in the pipeline [9,58,59,60]. Nevertheless, there are few overseas public reports on the liquid ultrasonic inspection of a pipeline girth weld crack defect. PetroChina Pipeline Company verified the reliability of this technology through the results of a traction test and actual in-line inspection, and its research achievements revealed that [51] ultrasonic in-line inspection technology could effectively detect the crack defects inside girth weld, especially cracks on the inner surface. The detection rate of girth weld crack defects in the simulation was larger than 90%, and the undetected crack defects mostly have the length of 30 mm and below and the depth of 1–2 mm. Nevertheless, the sizing model for this technology had not been perfected, so the defects could only be classified roughly in terms of their depth, and it was impossible to provide the specific size of defects. Defects were only classified into two categories, i.e., ≥2.5 mm and <2.5 mm. Now, the judgment is not highly accurate. For crack defects of lower depth, the judgment results are more inaccurate. However, the defects of higher depth (above 40 mm long, 4 mm deep) are more accurately identified.

Based on the results of liquid ultrasonic crack inspection for a pipeline, 42 defects were selected for excavation verification [22,24]. For 18 linear defects in the ultrasonic inspection report, excavation verification revealed lack of fusion of the girth weld at two positions, incomplete penetration at one position and no defect at three positions. Most defects identified in ultrasonic inspection belonged to geometrical defects, e.g., uneven surface, repair welding, undercut, and slag inclusion, etc. For the ultrasonic in-line inspection of pipelines, the defect identification based on ultrasonic waves is significantly affected by the interior wall of the pipe, air bubbles in motion, and geometry of the girth weld, which should be further studied.

### 3.3. Electromagnetic Acoustic Transducer Inspection of Natural Gas Pipeline Girth Weld Defects

EMAT technology follows the principle of electromagnetic induction eddy current to generate ultrasonic waves, and its converter can be kept separate from the surface of the inspected object, and does not require any coupling medium. In addition to its suitability for sulfide corrosion crack (SCC) crack detection, it will enjoy a bright future in the in-line inspection of oil and gas pipelines.

#### 3.3.1. Inspection Principle

**Figure 14:**
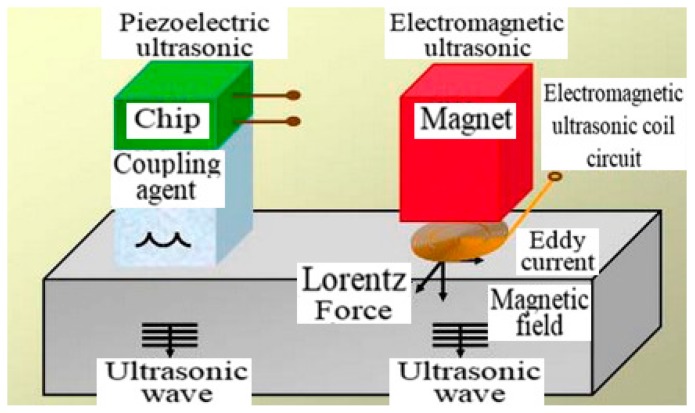
The comparison of electromagnetic and piezoelectric ultrasonic testing.

EMAT inspection technology is also known as eddy current-acoustic inspection technology, and its inspection principle is presented in Figure 14 [61]. When the coil with high-frequency current gets close to the pipeline, high-frequency eddy current will be inductively generated on the surface of the pipeline, resulting in the Lorentz force under the effect of the magnetic field. The collision of Lorentz force against metal lattice or another microscopic process is passed to the inspected material, creating a source of ultrasonic waves. The ultrasonic waves reflected from the defect of the pipeline form the eddy current under the effect of an external magnetic field, while the magnetic field generated by the eddy current leads to the change of voltage at both ends of the coil. Through analyzing the voltage signal, the defect can be located and classified. If the material is ferromagnetic, the dynamic magnetic field generated by the alternating current has an interaction with the magnetization intensity of the material under the influence of magnetostriction, which results in an ultrasonic coupling source.

Common ultrasonic and electromagnetic ultrasonic technology share many similarities (Figure 14) except that the generation is achieved by piezoelectric chip or electromagnetic induction. Electromagnetic ultrasonic technology utilizes the electromagnetic effect to generate an ultrasonic signal in the inspected metal pipe, which does not need a coupling agent due to direct contact, so it can directly inspect the pipeline with the settled layer or protective layer on the surface [62]. However, the electromagnetic ultrasonic signal has lower amplitude and higher sensitivity to the noise in the surroundings [63,64].

**Figure 15:**
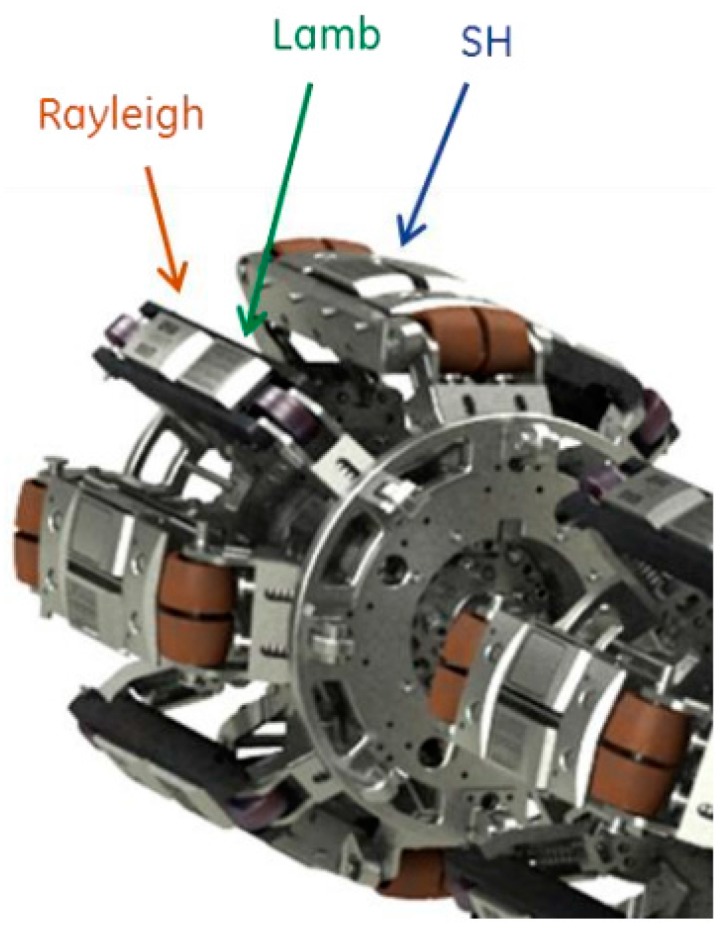
Electromagnetic Acoustic Transducer (EMAT) inline inspection tool sensor carrier and sensor types.

TransCanada, a Canadian company, started an attempt associated with PII to apply the EMAT technology in pipeline inspection equipment [65], and employed the EMAT sensors to generate the shear horizontal wave (SH wave), surface wave (RH wave) and Lamb wave for measurement (Figure 15). The SH wave is a horizontal polarization shear wave, which travels inside the pipe wall and can be used to detect any crack and size the crack depth. The SH wave has some special advantages. When it is reflected at the boundary, the reflected wave does not cause the conversion of waveform, but contains only the SH wave. When it is used in measurement, the signal analysis is simplified. The surface wave (RH wave) travels only in the inner surface of the pipe wall and can be used to identify the defects of inner and outer surfaces. The Lamb wave transmits into the pipe wall directly, and travels circumferentially inside the pipe, so it can be used to measure the wall thickness of the pipe, and distinguish cracks from no damage. Now, this technology has been proved to be effective to detect the stress corrosion crack and longitudinal weld crack of gas pipelines [66,67,68]. GE PII and Rosen have developed the mature in-line inspection equipment [69,70], and some Chinese universities have been engaging in studies on the technology and equipment of EMAT inspection for pipeline defect, including Tianjin University [71], Shenyang University of Technology [72,73], Beijing University of Chemical Technology [74] and Tsinghua University [63].

**Figure 16:**
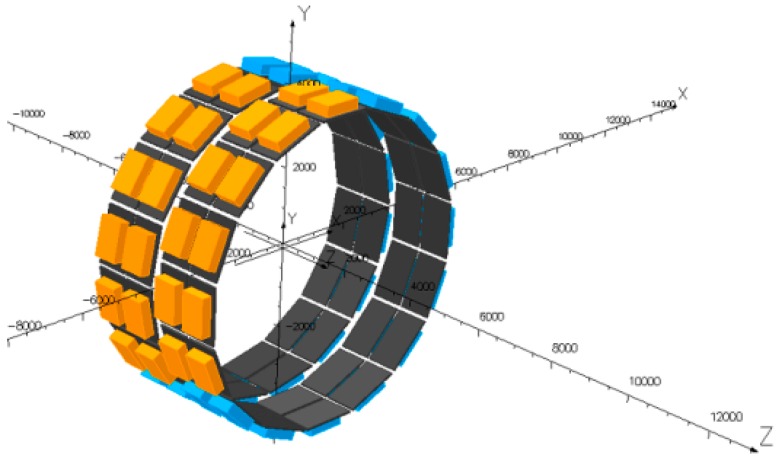
Allocation of EMAT inspection sensors for circumferential defects (unit: mm).

The inspection of a girth weld crack is similar to that of a longitudinal weld crack, but it is relatively more difficult to inspect the circumferential crack. PetroChina Pipeline Company and PII carried out the feasibility study on the EMAT inspection technology and equipment for pipeline girth weld defects, and employed the axially arranged EMAT converter based on the characteristics of a pipeline girth weld defect [75]. To guarantee the full circumferential coverage of pipeline girth weld, pairs of EMAT converters are distributed along the circumference (Figure 16) to solve the problem of potential noise caused by the number of collection channels and the crosstalk between converters. Moreover, sufficient counts per revolution must be realized, and a minimum crack length of the inspected object is utilized to optimize the width of the EMAT converters.

#### 3.3.2. Signal Recognition and Defect Sizing

Now, international studies on electromagnetic ultrasonic signal focus on the suitable signal processing methods, such as, wavelet conversion, to eliminate noise and identify the defect. The band-pass filter based on discrete Fourier transform can be utilized to effectively eliminate electronic noise, but this method becomes ineffective when the frequency of the input signal varies with time, and loses the information on time domain of signal peak [76]. The time frequency signal processing method, e.g., nonlinear adaptive filter, has very good performance to deal with nonlinear and non-stationary problems, but its structure is complicated, and its sensitivity depends much on initial parameters [77]. The receiving signal of electromagnetic ultrasonic technology can be regarded as a non-stationary signal due to the overlapping of noise, while the amplitude and phase, etc., among sine components under different frequencies are time-varying parameters. EMAT signal processing means to extract the single sine signal from the given multi-component input signals [78,79]. To extract a non-stationary signal, reference [80] proposed a nonlinear and adaptive time-domain signal processing method based on traditional Fourier transform.

Yang Han et al. [81] demarcated the EMAT echo signal based on the size of a known defect to obtain the corresponding relationship between the echo signal and size of a crack-like defect, and utilized the suitable data analysis method to calculate the depth and length of a crack, so as to realize the sizing of the crack defect. PetroChina Pipeline Company (Langfang, China) carried out the study of pipeline girth weld defect inspection together with GE Oil & Gas Pipeline-inline-inspection Solutions (GE PII, Newcastle, UK) [75] to reveal that the time of flight of the incident wave could not be a sufficient factor for the identification of the girth weld notch, but the data analysis based on signal amplitude might be conducted to detect and identify the girth weld notch. The girth weld defect inspection and sizing based on signal amplitude should be further studied.

#### 3.3.3. Inspection Reliability

Based on the problem of girth weld crack defect inspection for long-distance natural gas transport pipelines, PetroChina Pipeline Company(Langfang, China) is cooperating with GE Oil & Gas Pipeline-inline-inspection Solutions (GE PII, Newcastle, UK) to conduct the preliminary study on the EMAT inspection technology of girth weld crack defects. Nevertheless, the reliability of EMAT inspection for girth weld crack defects has not been publicly reported. Hence, the reliability of EMAT inspection for pipeline axial cracks is introduced for reference.

TransCanada (Calgary, AB, Canada) Pipeline generalized the data of electromagnetic ultrasonic inspection for axial cracks and stress corrosion cracks (SCC) in 13 pipelines from 2008 to 2011 [82]. The results revealed that the EMAT detection rate of axial cracks exceeded 90%, and the success rate of EMAT inspection for the depth of a pipeline axial crack ranged 86%–100%, and improved from 86% in 2008 to 100% in 2011. Nevertheless, the length involved in in-line inspection was the total length of the crack subject to crack cluster or interaction, so it was more difficult to determine the length of the crack than the depth. The success rate of inspection for the length of axial SCC was 0%–33%. The length of the crack is not very sensitive to integrity, but further work should be carried out to analyze and improve the accuracy of inspection.

Kinder Morgan studied the reliability of EMAT inspection for axial cracks of pipelines [83]. The EMAT inspection had the defect depth measurement ±18%, certainty of 80% and confidence level of 90%. The EMAT inspection was highly accurate and increased along with defect depth, but the inspection results tended to be overestimated. Any 2 mm deep and 40 mm long crack could be highly identified, but any blunt crack with a depth of 1–2 mm could not be highly identified. The inspection results were not reliable for cracks of less than 1 mm before the surface.

In 2008, Pipeline Research Council International (PRCI) initiated a program for assessment of EMAT in-line inspection performance, and employed the technologies including magnetic particle, ultrasonic inspection, phase-array ultrasonic imaging and crack interruption to compare the size of a pipeline defect from EMAT in-line inspection with the data of field excavation inspection [84]. After comparing the EMAT and non-destructive evaluation (NDE) data of the crack surface for one crack, the crack outline was consistent in both inspections, and the maximum crack depth was within the deviation ±1 mm. When the actual crack depth was 2.7 mm, the size from the EMAT inspection was 1.1 mm larger than the actual defect depth, and slightly higher than the accuracy range of NDE (−0.2 mm–0.8 mm). The crack length deviation at the maximum depth was within 10%, but the length in the EMAT in-line inspection report was the length of the crack cluster, instead of the length of the deepest crack.

### 3.4. Electromagnetic Eddy Current Inspection

In recent years, remote field eddy current (RFEC) inspection has been an important technology for natural gas pipeline girth weld crack defect inspection. Reference [85] reported that the accuracy of Russell Corporation’s RFEC depth inspection for a natural gas pipeline crack could reach 5%. Now, the application of this technology in natural gas pipeline crack inspection has attracted attention from domestic and overseas pipeline operation companies and inspection companies.

#### 3.4.1. Inspection Principle

**Figure 17:**
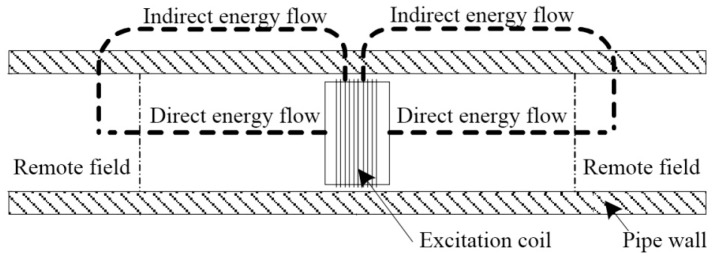
Schematic diagram of the electromagnetic eddy current inspection principle.

Electromagnetic eddy current in-line inspection technology can effectively receive the magnetic field that passes the tube wall and returns to the inside tube by employing the excitation coil or placing a measurement coil with a certain distance from the excitation coil, so as to effectively detect the interior wall defect of the metal tube. The inspection principle is as shown in Figure 17 [86]. The inspection coil of RFEC is located in the “remote field area” with a distance equivalent to 2~3 times of the tube’s inside diameter from the excitation coil. In this area, the amplitude and phase of the electromagnetic eddy current signal decrease at a very low speed, which is the same inside and outside the tube. The phase delay of induced electric potential is roughly directly proportional to the thickness of the tube wall that it passes through, and it can be calculated approximately by employing the phase formula for the one-dimensional skin effect for calculation [87].

Unlike the common eddy current testing which is the impedance plane analysis form, the remote field eddy current (RFEC) technique reduces problems such as lift-off. As the excitation source frequency is generally low, RFEC does not have the skin effect, and the RFEC signal is not sensitive to the lift-off effect. With the same inspection sensitivity to crack defects on the inner and outer surfaces of pipelines, it can effectively overcome the limitations of the common eddy current inspection method, so it is more suitable for the inspection of natural gas pipeline surface cracks. JENTEK Sensors Inc., a U.S. company, has developed the electromagnetic eddy current technology with a magnetic winding meter (MWM) array to improve the identification of imperfections sensitive to lift-off value, which has been proved to be suitable for the identification and sizing of axial cracks in oil and gas pipeline. In the stress corrosion crack inspection, the technology has been verified to excel other inspection methods in the possibility of detection (POD), lift-off value modification and speed modification, etc. [88]. RFEC inspection has the same sensitivity to the defects in the interior and exterior walls of pipeline, but it is still a technical problem to distinguish these defects [89]. Yang Lijian et al. [90,91] in Shenyang University of Technology designed and optimized the RFEC inspection system based on simulation analysis, and distinguished the defects in the interior and exterior walls of pipeline based on the amplitude of the output voltage signal.

The RFEC technology with the traditional sine excitation also has some limitations, such as, weak signal, slow speed and low rate, and the sine excitation signal causes very high power consumption at sensors [92,93]. Based on the problems in the traditional RFEC technology, pulsed RFEC technology has been developed recently, which allows one to place the inspection coil in the transitional area closer to the excitation coil. In this new technology, the detection signal is the superimposition of the pulsed magnetic field component and the eddy current magnetic field component, which can realize the effect of the far-field eddy current detection, and the detection signal amplitude increases, which is advantageous for the signal characteristic extraction. Shenyang University of Technology [94] and National University of Defense Technology [95] have carried out some studies on the pulsed RFEC inspection equipment, but RFEC inspection is mainly applied in the inspection of pipeline axial cracks at present.

#### 3.4.2. Defect Sizing Method

Electromagnetic RFEC inspection is carried out to detect imperfections based on the signal phase difference rather than signal amplitude, and the phase difference between signals has an approximate linear relationship with defect depth [96]. Wu Dehui et al. [97,98] carried out the systematic finite element simulation for the magnetic field of the RFEC defect in pipeline, and concluded that there was an exponential relationship between defect depth and phase of inspection signal, and a logarithmic relationship between defect width and phase of inspection signal. Liu Chunyan [99] performed the simulation analysis on the RFEC inspection of axial and circumferential cracks and pitting defects, employed the phase difference and amplitude variation curve to represent the symmetrical defects in the voltage planar polar diagram, and provided the basis for defect shape restructuring. Zhang Wei et al. [100] put forward a waveform approximation technique based on minimization criterion of mean-square error to characterize the waveform of the inspection signal at high accuracy. By building a nonlinear polynomial inversion model, a Back-Propagation (BP) Neural Network inversion model based on one-dimensional search and optimization, and a support vector machine inversion model based on particle swarm search and optimization, they realized the quantification inversion from the waveform characteristics of inspection signal to the size of crack defect. The relative deviation of calculation with the inversion model was lower than 5%, and the model had a strong ability of interference resistance, so it was a powerful tool for quantification inversion of the crack defect. Liu Hongqing [101] conducted the systematic finite element simulation for the pipeline RFEC defect magnetic field, and utilized a three-dimensional model to simulate the dent and crack defects that were not axially symmetric, so as to obtain three signal characteristics of dent crack and axial crack defect in different directions. Also, Liu analyzed how the signal characteristics of an axial defect related to its circumferential width, axial length and radial depth.

For defect sizing in pulsed RFEC inspection, Yang Binfeng et al. [102,103] and Yang Lijian et al. [104] analyzed the RFEC inspection mechanism under pulse excitation, and took the zero-crossing time in the inspection signal as the characteristic value of defect sizing. The experiment was conducted to verify the quantitative assessment of length and depth for axial crack defects in the pipeline with the RFEC technology under pulse excitation, and effectively distinguish the defects in the full circumference inside and outside the pipe wall.

RFEC inspection technology can detect the defects on the surface of natural gas pipelines without being significantly affected by lift-off and eccentricity, etc. Nevertheless, the RFEC inspection signal is weak and slow, so its overall inspection efficiency is very low. For this reason, RFEC technology should be further studied in the future.

## 4. New Technologies for Pipeline Defect In-Line Inspection

To improve the accuracy of pipeline defect inspection, domestic and overseas science and technology personnel are constantly exploring the new pipeline defect inspection methods, including Pulse echo (PE)/TOFD composite inspection technology, laser ultrasonic, magnetostriction, and other contactless inspection technologies, which have great potential of application in the long-distance transport pipeline girth weld defect inspection. Hence, these technologies are briefly described in the following sections.

### 4.1. Composite Ultrasonic Inspection

**Figure 18:**
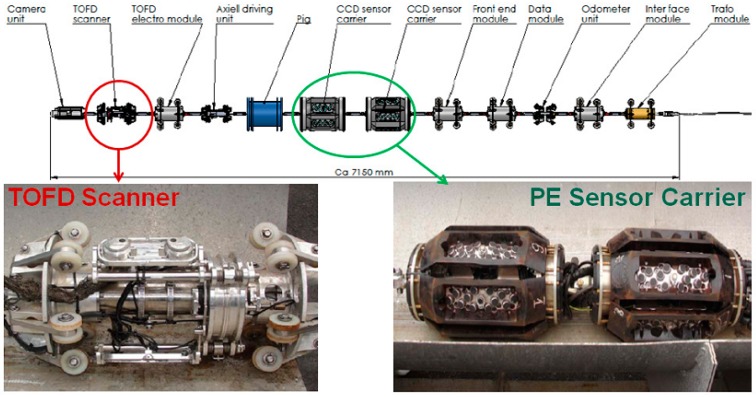
Setup of the 10° tethered inspection tool (**top**). (Lower **left**) TOFD scanner; (Lower **right**) Sensor carrier for pulse-echo inspection.

Statoil [105] has developed a new composite ultrasonic inspection technology to detect the girth weld crack defects of submarine pipeline, i.e., pulse-echo (PE)/TOFD composite inspection. This technology consists of a TOFD inspection unit and a PE inspection unit (Figure 18), which combine the rapid detection of PE inspection with the crack defect sizing of TOFD. For the crack defect, the depth inspection accuracy is 1 mm, the minimum length of a crack is 10 mm and the detection rate is 90%, which satisfy the expectations of industrial application. TOHO, a Japanese company [106] and Tianjin University [107] have taken the composite aperture focusing ultrasonic imaging technique as an ultrasonic processing method, and employed the point-by-point focusing method to achieve no variation of image resolution with location and depth. When the same array element sensor carrier of converter is employed, the composite aperture focusing method is utilized to obtain the rebuilt images with higher resolution, so as to provide a more reliable basis for the characterization and sizing of a defect.

### 4.2. Laser Ultrasonic Inspection

Laser ultrasonic inspection is a new technology, popular in recent years. Ultrasonic waves are generated on the surface of the inspected work piece by pulsed laser beam irradiation. After changing the experimental parameters, the laser ultrasonic source can induce many kinds of guided waves, including longitudinal wave, lateral wave and surface wave. The ultrasonic signal can be generated by laser excitation and detected by the optical method, so it can realize completely contactless inspection and quick scan imaging, and facilitate the realization of nondestructive testing under adverse conditions, e.g., high temperature and strong vibration [108]. Additionally, a mode-locking laser can be utilized to more easily obtain the ultrasonic pulse with the width similar to laser pulse, and its frequency band is much wider than the ultrasonic wave generated by a common converter. Hence, the defect inspection technology based on ultrasonic diffraction is very sensitive to the tiny cracks on the surface and near the surface of the inspected work piece, and has higher inspection accuracy than other nondestructive testing techniques [109]. APPLUS RTD has utilized the one-time laser ultrasonic inspection technique in the TOFD technology to measure the depth of SCC reliably and accurately. Moreover, this technology is also suitable for measuring the depth of other cracks, e.g., crack abnormalities [110]. Intelligent Optical Systems has successfully applied this technology in the inspection of pipeline girth weld detects [111].

### 4.3. Magnetostriction Inspection

Magnetostriction is an innate feature of ferromagnetic materials. The magnetostriction effect and its reverse effect can be utilized to generate and receive the ultrasonic guided waves inside ferromagnet. A magnetostriction sensor can be utilized to detect the crack, corrosion and other defects of wire rope, metal rod, pipe and plate [112,113]. Even if the air gap between the inspection probe and inspected pipe surface is relatively large, the magnetostriction sensor can also transmit and detect the guided waves, and themagnetostriction effect is very strong at the end of a crack. Thanks to the good characteristics of transmission and the same inspection sensitivity to defects on the inner and outer surfaces of pipeline, more and more nondestructive testing scholars at home and abroad have paid attention to the study on the application of magnetostriction in the quick inspection and crack identification of pipelines in recent years.

## 5. Conclusions and Prospects

This paper analyzes the characteristics of long-distance transport pipeline girth weld defects, and summarizes the inspection principle, defect signal identification, defect sizing method and inspection reliability of existing girth weld defect in-line inspection technologies systematically, as well as the new technology of pipe defect in-line inspection. It is comprehensively concluded that:

1. Three-axis high-resolution MFL inspection technology can identify a lot of long-distance transport pipeline girth weld defects. It is highly adaptive and able to effectively detect the volumetric defects, but the accuracy of defect sizing is not high.
2. Neither three-axis high-resolution MFL inspection nor liquid ultrasonic inspection can effectively detect the closed crack defect of long-distance transport pipeline girth weld, except the defects with a large opening (about >0.5 mm).
3. The future development will focus on improving the existing electromagnetic ultrasonic crack inspection and RFEC inspection, developing and applying new crack inspection technology and realizing the effective inspection of girth weld crack defects.
4. Due to the complexity of the on-line inspection condition and the principle of detection technologyfor long-distance transport pipeline girth weld, it is very difficult to detect and quantify the girth weld defects. The irregular outline of girth weld, surface condition of pipeline and other factors have inevitable influence on the inspection and sizing of girth weld defects, and attention should be paid to the actual pipeline inspection factors during inspection data analysis and sizing.
5. At present, the finite element method and the neural network method are used to quantify the defects of girth welds, but the accuracy is far from satisfying the requirements. The type and sizing of defects need to be studied systematically.
6. In order to improve the accuracy of pipeline defect inspection, the new technologies for contactless inspection have been applied in the in-line inspection of pipeline defects, including composite inspection, laser ultrasonic and magnetostriction.
